# A Systematic Evaluation of Risk Predictors for COVID-19 Sequelae

**DOI:** 10.7759/cureus.40717

**Published:** 2023-06-21

**Authors:** Harshmeet Singh Gujral, Tushar R Sahasrabudhe, M A Nirmala

**Affiliations:** 1 Respiratory Medicine, Dr. D. Y. Patil Medical College, Hospital and Research Centre, Dr. D.Y. Patil Vidyapeeth, Pune, IND; 2 Respiratory Medicine, Dr. D. Y. Patil Medical College, Hospital and Research Centre, Dr. D. Y. Patil Vidyapeeth, Pune, IND; 3 Respiratory Medicine, Aster Medcity, Bangalore, IND

**Keywords:** pulmonary fibrosis, risk predictors, multisystem, sequelae, covid

## Abstract

Background

Multisystem involvement in coronavirus disease 2019 (COVID-19) is known since the beginning of the pandemic, and post-COVID-19 sequelae have often been reported. The term 'long Covid' encompasses these signs and symptoms. The aim of our study was to study different after-effects which patients endured within 12 months after recovery from acute COVID-19 and to study the various risk predictors.

Methods

This was a longitudinal observational study of a cohort of 146 patients who recovered from COVID-19 illness. Patients were enrolled during the first four weeks of the onset of their illness, and a monthly follow-up assessment was done for six months that included a detailed history of persistent or new symptoms, new illnesses diagnosed, and complete biochemical, pulmonary, cardiac, neurological and psychiatric evaluation, both objective and subjective. A final follow-up was also done at the end of one year of enrolment. Based on the patient’s self-reported history and our multi-system assessment, recorded sequelae were classified according to the involved organ system. These were correlated with possible risk predictors and statistically significant associations were established.

Results

One hundred and twenty subjects out of 146 total subjects qualified for final analysis. Pulmonary sequelae (48/120; 40%) were the most followed by psychiatric (30/120; 25%), neurological (26/120; 21.7%), and opportunistic infections (7/120; 5.8%). A total of 39/120 (32.1%) cases complained of prolonged dyspnoea. Six out of 120 i.e. 5% of study participants had new-onset diabetes. Twenty-six out of 120 (21.7%) had radiological signs of pulmonary fibrosis. Patients with co-morbidities, older age, higher body mass index, and patients with severe disease were found to be at higher risk of developing these sequelae. Poor nutrition, female gender, and hospitalization were predictors of psychiatric sequelae. Diabetes and liberal steroid use during COVID-19 management were predictors of opportunistic fungal infections.

Conclusion

This study evaluated post-COVID-19 sequalae in-depth both objectively and subjectively. Some specific predictors for specific sequelae were confirmed on statistical correlation. Long-term follow-up of high-risk persons is therefore recommended after the cure of COVID-19.

## Introduction

Coronavirus disease-2019 (COVID-19), hereafter abbreviated as only “Covid” was declared a pandemic on March 11, 2020, because of the worldwide impact it had on overall morbidity and mortality [1]. Although it primarily affects the respiratory system, Covid is a systemic disease with multi-organ involvement. With increasing age and co-morbidities, the risk of developing a severe disease increases manyfold, and the viral illness may last up to four weeks [2]. Even after recovery from Covid, significant respiratory, neuropsychiatric, circulatory, and musculoskeletal dysfunction may still persist [3,4].

In addition to the virus itself, the body's reaction to the virus, the effects of drugs, and the psychosocial repercussions of the distress patients underwent during the pandemic, all left a profound impact on the patient's well-being. In addition, associated psychosocial factors may have had a significant impact on mental health [5]. The terminology "long Covid" is being used to address this wide array of symptomatology that persist or worsen after four weeks post-acute Covid infection. New symptoms or illnesses may emerge after four weeks which may be directly or indirectly related to Covid. These and the persistent post-Covid symptomatology have been termed "post-COVID-19 syndrome" [6].

The objective of this study was to investigate the various sequelae or after-effects that patients experienced post-Covid, in the first 12 months following the resolution of acute disease, as well as to study any potential risk factors/predictors linked to these sequelae.

## Materials and methods

This was a longitudinal observational study conducted in a dedicated Covid hospital in Pune, Maharashtra, India, in the years 2021 and 2022. The study commenced after the approval by the Ethics sub-committee at Dr. D.Y. Patil Medical College with approval number L.E.S.C/153/2021. The minimum required sample size was 63. The estimation was done on the basis of the prevalence of neurological sequelae in Covid in 2020, at the time of the commencement of the study, which was 36.4% with an acceptable difference of 12% (WinPepi software, version 11.65 {www.brixtonhealth.com/pepi4windows.html} was used for sample size estimation). Patients above 18 years of age, suffering from lab-confirmed acute Covid were enrolled during the first four weeks of the onset of acute Covid symptoms, but after they started recovering from their acute illness. A confirmed Covid case was defined as a person whose nasopharyngeal swab tested positive for severe acute respiratory syndrome coronavirus 2 (SARS-CoV-2) by RT-PCR (quantitative real-time reverse transcription polymerase chain reaction), and recovery from acute infection was defined as the time after four weeks from the symptom onset of Covid [7]. For analytical purposes, the enrolled participants were categorized based on the severity of their disease (as per the guidelines defined by the Indian Council of Medical Research) [7]. Exclusion criteria included pregnancy, language barrier, and inability to perform required assessment tests as per the study protocol. Termination criteria included death, relocation, and failure to adhere to the protocol.

A total of 146 patients were enrolled. Patient demographics, data on Covid related symptomatology, laboratory/radiological investigations, and past medical history were captured. All the potential risk predictors for specific sequelae such as age, sex, body mass index (BMI), socioeconomic status, family background, pre-existing co-morbidities, the severity of the disease, smoking history, need for hospitalization, etc. were assessed and correlated with these sequelae (please refer to the Results section). These subjects were tracked and closely followed up for a total of six months via telecommunication and an in-person visit was scheduled every month at the patient’s convenience, for the purposes of pulmonary assessment (recording symptoms, clinical examination, chest imaging, six-minute walk test, and spirometry), neurological assessment (recording neurological symptoms, clinical examination including higher functions, sensory-motor function, deep tendon reflexes nerve conduction study, and radiological assessment when indicated), cardiology assessment (clinical examination, 12-lead ECG, treadmill stress test and echocardiography and angiography wherever indicated) and psychiatric assessment. Mini International Neuropsychiatric Interview (M.I.N.I.) version 5.0.0 questionnaire was used for psychiatric evaluation. Enrolled participants had no prior history of mental illness. Biochemical workup included blood sugar, HbA1c levels, hemogram, renal function tests, liver function tests, and inflammatory markers. Any secondary infections that occurred during the first four weeks of Covid were considered as a complication and not as a sequela. After a year of enrolment, patients were contacted by phone once more to obtain a final update on the post-covid sequelae and their general well-being.

MedCalc (MedCalc Statistical software version 18.2.1 (MedCalc Software bvba, Ostend, Belgium) and WinPepi software were used for the processing and analysis of the data. Frequencies and proportions were used to express categorical variables. To investigate the relationship between categorical variables, the relative risk was calculated with 95% confidence intervals. Statistical significance was defined as a p-value of less than 0.05. To investigate the relationship between continuous variables like age and BMI, Cohen’s D test was used to calculate effect size. The effect size was defined as small (d = 0.2-0.5), medium (d = 0.5-0.8), and large (d > 0.8).

## Results

**Figure 1:**
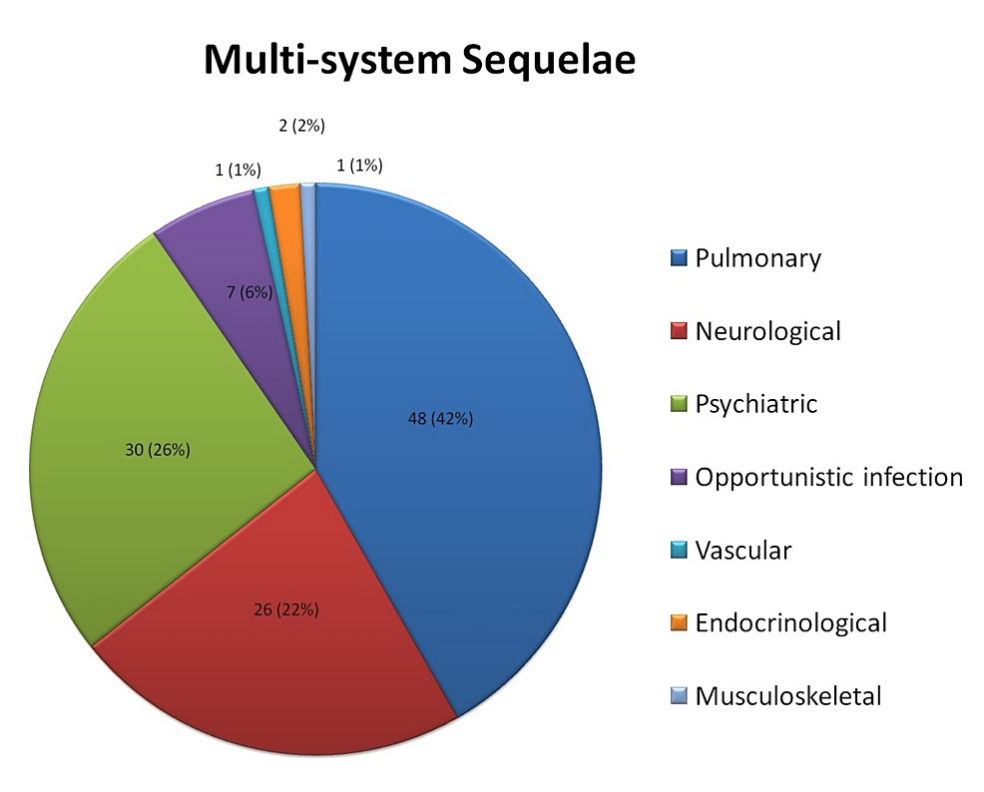
Pie chart depicting the distribution of various sequelae

Out of the 146 enrolled subjects, 16 were lost to follow-up and 10 revoked consents during the study period. Data from the remaining 120 patients was analysed. The age of participants ranged from 20 to 83 with the median age being 41 years and 76/120 (63.3%) were males. With respect to occupation, healthcare workers (43/120, 35.8%), followed by service industry workers (23/120, 19.2%) were the common occupations whereas 24/120 (20%) were unemployed. Thirteen out of 120 (10.8%) participants were smokers and 44/120 (36.6%) participants had pre-existing co-morbidities, the majority being diabetes mellitus (16/120, 13.3%), bronchial asthma (15/120, 12.5%) and hypertension (10/120, 8.3%). In-patient care was required for 72/120 (62%) participants during the acute phase of Covid. 51/120 (43%) participants had moderate or severe disease. With regards to treatment, systemic corticosteroids for minimum 10 days (usual starting doses were intravenous methylprednisolone 40 mg twice daily or oral dexamethasone 6 mg once daily) were prescribed in 46/120 (38.3%) cases, 31/120 (25.8%) participants received remdesivir, 66/120 (55%) received anticoagulants (0.5 mg/Kg enoxaparin as prophylaxis for at least seven days) and 19/120 (15.8%) patients needed intensive care for Covid treatment. None of the enrolled subjects had confirmed pulmonary thromboembolism during Covid and hence therapeutic doses were not used for any of the study participants. The median duration of hospital stay was 17 days ranging from three to 50 days. Sequelae reported by the patients at the end of the follow-up period were classified based on the involved organ system (Figure 1).

Out of 120 study participants, 74 (61.6%) had functional or structural post-covid sequelae of some kind. The majority (48/120 i.e., 40%) of sequelae were pulmonary, followed by psychological (30/120 i.e., 25%), neurological (26/120 i.e., 21.7%), and infection from opportunistic pathogens (7/120 i.e., 5.8%). The risk association of various sequelae with categorical variables have been tabulated below (Tables 1-4) for pulmonary, psychological, and neurological sequelae respectively. The strength of the association between various sequelae and continuous variables like age and BMI have been tabulated in Table 4.

**Table 1:** Risk predictors for pulmonary sequelae (for categorical variables) RR: Relative risk Statistically significant findings are highlighted in bold letters.

	Risk Predictors	Relative risk and P value									
		Pulmonary sequelae (Combined)		Prolonged Dyspnoea		Pulmonary fibrosis		Prolonged Cough		Pneumothorax	
		RR	P value	RR	P value	RR	P value	RR	P value	RR	P value
1.	Male Sex	1.0132	P=0.95	1.47	P=0.19	1.5714	P=0.2579	0.69	P=0.52	0.28	P = 0.30
2.	Smoking history	0.823	P=0.654	1.210	P=0.613	0.68	P=0.576	0.82	P=0.84	1.10	P=0.947
3.	Co-morbidities (combined)	1.7273	P=0.019	2.01	P=0.006	2.355	P=0.014	2.07	P=0.20	0.86	P = 0.90
a.	Diabetes mellitus	1.6714	P=0.048	1.95	P=0.01	2.88	P=0.001	0.65	P=0.67	0.88	P = 0.93
b.	Hypertension	2.3123	P<0.001	2.673	P<0.001	2.91	P=0.002	2.71	P=0.153	6.11	P = 0.12
c.	Heart disease	0.6742	P=0.748	0.75	P = 0.82	1.13	P=0.92	2.60	P=0.44	8.57	P=0.107
d.	Allergic Rhinitis / Asthma	0.3302	P=0.09	0.37	P=0.14	0.27	P=0.19	0.69	P=0.71	0.93	P = 0.96
4.	Moderate/ Severe disease	5.8846	P<0.001	8.89	P<0.001	69.00	P=0.0028	1.56	P=0.43	2.61	P=0.42
5.	Positive Pressure Ventilation	11.97	P=0.09	1.80	P=0.058	3.65	P<0.001	2.20	P=0.26	19.8182	P=0.001

**Table 2:** Risk predictors for psychological sequelae (for categorical variables) RR: Relative risk; GAD: Generalised anxiety disorder; PTSD: Post-traumatic stress disorder Statistically significant findings have been highlighted in bold letters.

	Risk Predictors	Relative risk and P value									
		Psychological sequelae (Combined)		Depression		GAD		Dysthymia		PTSD	
		RR	P value	RR	P value	RR	P value	RR	P value	RR	P value
1.	Female Sex	1.957	P = 0.024	1.72	P = 0.696	2.59	P = 0.12	2.015	P = 0.18	1.72	P = 0.42
2.	Smoking history	0.85	P = 0.76	1.542	P=0.773	2.05	P = 0.32	0.685	P = 0.70	0.4538	P = 0.57
3.	Comorbidities	1.036	P = 0.90	0.342	P = 0.48	0.740	P = 0.65	0.31	P = 0.12	12.09	P = 0.017
a.	Diabetes mellitus	0.92	P = 0.87	1.2353	P = 0.88	0.294	P = 0.39	0.22	P = 0.29	6.50	P = 0.004
b.	Heart disease	0.92	P = 0.94	12.00	P=0.07	2.857	P = 0.40	2.22	P = 0.52	0.28	P = 0.32
c.	Allergic Rhinitis/Asthma	0.9905	P = 0.9833	1.31	P = 0.85	2.97	P = 0.08	0.57	P = 0.58	0.386	P = 0.50
4..	Moderate/ Severe disease	1.9112	P = 0.03	0.26	P = 0.38	1.961	P = 0.27	1.12	P = 0.82	22.13	P = 0.03
5.	Hospitalization	6.009	P = 0.001	0.62	P = 0.73	5.59	P = 0.096	7.45	P = 0.049	10.65	P = 0.10
6.	ICU stay	3.189	P<0.001	1.02	P = 0.98	0.59	P = 0.607	2.36	P = 0.11	86.70	P = 0.001
7.	Steroid use	1.82	P=0.04	0.31	P=0.45	1.07	P=0.90	1.37	P=0.53	27.12	P=0.022

**Table 3:** Risk predictors for neurological sequelae (for categorical variables) RR: Relative risk Statistically significant findings have been highlighted in bold letters.

	Risk Predictors	Relative risk and P value							
		Neurological sequelae (Combined)		Persistent Ageusia		Persistent Anosmia		Persistent Headache	
		RR	P value	RR	P value	RR	P value	RR	P value
1.	Female Sex	1.46	P = 0.29	1.1579	P = 0.86	0.987	P = 0.982	2.303	P = 0.25
2.	Smoking	0.74	P = 0.66	1.542	P=0.773	1.8291	P = 0.4046	0.5143	P = 0.6425
3.	Comorbidities	1.727	P = 0.13	0.86	P = 0.86	0.9870	P = 0.9825	0.6909	P = 0.6500
a.	Diabetes mellitus	1.30	P = 0.58	0.475	P = 0.60	0.2685	P = 0.3548	1.0833	P = 0.9390
b.	Hypertension	0.53	P = 0.51	0.85	P = 0.91	0.4826	P = 0.6044	0.7400	P = 0.8324
c.	Allergic Rhinitis/ Asthma	1.38	P = 0.48	2.673	P<0.001	3.9619	P = 0.0144	0.4375	P = 0.5648
d.	Moderate/ Severe disease	1.30	P = 0.46	1.3077	P = 0.735	0.4904	P = 0.2740	1.7436	P = 0.4532
4.	Remdesivir use	0.75	P = 0.53	0.21	P = 0.2921	0.1223	P = 0.1417	3.8280	P = 0.06

**Table 4:** Risk association for continuous variables with sequelae Cohen’s D test was used to calculate effect size to categorize the strength of association between the development of sequelae and risk predictors like age and BMI. The effect size was defined as small (d = 0.2-0.5), medium (d = 0.5-0.8), and large (d > 0.8). A negative sign means group 2 has a greater value than group 1.

Sequelae	Age				BMI			
Outcome	Present	Absent			Present	Absent		
	Mean +/- SD		Cohen's D	Effect size	Mean +/- SD		Cohen’s D	Effect size
Pulmonary sequelae (combined)	53.32 ±18.32	34.95 ±15.44	1.0843	Large	25.87 ±2.91	25.15 ±1.75	0.299	Small
Pulmonary Fibrosis	58.38 ±16.33	37.06 ±16.65	1.2928	Large	26.54 ±2.91	25.09 ±1.94	0.5863	Medium
Persistent dyspnea	55.72 ±18.39	34.92 ±14.78	0.5515	Medium	25.80 ±2.74	25.22 ±1.97	0.2431	Small
Pneumothorax	41.33 ±20.55	41.69 ±18.77	-0.0183	Small	24.75 ±1.69	25.43 ±2.27	-0.3398	Small
Persistent cough	47.09 ±17.00	41.13 ±18.87	0.3319	Small	24.33 ±3.35	25.51 ±2.13	-0.4204	Small
Psychiatric sequelae (Combined)	40.75 ±15.28	42.02 ±19.89	-0.0716	Small	25.48 ±2.62	25.39 ±2.13	0.0377	Small
Depression	28.50 ±2.12	41.90 ±18.80	-1.0017	Large	24.18 ±0.22	25.47 ±2.27	-0.7999	Medium
Gen. Anxiety Disorder	32.90 ±10.70	42.48 ±19.12	-0.6183	Medium	24.17 ±1.28	25.51 ±2.30	-0.72	Medium
Dysthhymia	41.86 ±17.17	41.66 ±18.97	-0.011	Small	26.32 ±2.52	25.30 ±2.21	0.4304	Small
PTSD	54.75 ±10.01	40.75 ±18.87	0.9269	Large	26.52 ±3.87	25.33 ±2.10	0.3822	Small
Neurological Sequuelae (Combined)	42.66 ±17.91	41.43 ±19.00	0.0666	Small	25.75 ±2.95	25.32 ±2.06	0.169	Small
Persistent Aguesia	31.66 ±7.63	42.21 ±19.09	-0.7257	Medium	25.77 ±3.66	25.37 ±2.18	0.128	Small
Persistnt Anosmia	39.36 ±17.57	41.91 ±18.89	-0.1398	Small	24.86 ±1.56	25.42 ±2.32	-0.2833	Small
Persistent headache	38.00 ±10.98	41.91 ±19.10	-0.251	Small	25.43 ±3.31	25.41 ±2.20	0.0071	Small
Mucormycosis	54.14 ±17.65	40.91 ±18.58	0.7301	Medium	28.54 ±2.06	25.21 ±2.13	1.1593	Large

Many patients experienced persistent respiratory symptoms on follow-up. Out of the total, 32.5% (39/120) and 9.2% (11/120) of the participants reported complaints of persistent dyspnoea and chronic cough respectively. Radiological evidence of pulmonary fibrosis was present in 21.7% (26/120) of patients out of which 25 patients had complaints of persistent dyspnoea. During the follow-up period, 2.5% (3/120) of subjects developed spontaneous pneumothorax, necessitating tube thoracostomy. None of the study participants had pneumothorax during their acute Covid state (first four weeks from the symptom onset of Covid). Pleural effusion and pulmonary thromboembolism were diagnosed in 2/120 (1.6%) and 1/120 (0.8%) of the participants, respectively. Out of the 48 participants who reported pulmonary sequelae, 38 (79.2%) had moderate to severe Covid and 10 (20.8%) participants had a milder form of the disease. Radiologically evident pulmonary fibrosis was documented in 26 out of the 48 participants (54.16%) experiencing pulmonary sequelae. Twenty-four out of 26 (92.3%) had severe disease on presentation, and 2/26 (7.7%) had moderate disease. These sequelae were correlated with possible associated risk factors like sex, the severity of disease, co-morbidities smoking history, use of positive pressure ventilation, etc. and the results have been tabulated in Tables 1, 4. On spirometry, 28/120 (23.3%) cases showed restriction and 8/120 (6.7%) showed obstruction.

Among psychological sequelae, a total of 2/120 (1.7%), 10/120 (8.3%), 13/120 (10.8%), and 8/120 (6.7%) patients developed depression, generalised anxiety disorder (GAD), dysthymia, and post-traumatic stress disorder (PTSD) respectively as per M.I.N.I. 5.0.0. screening tool. Out of 30 participants who developed psychological sequelae, 17 (56.7%) were females and 13 (43.3%) were males. Nineteen out of 30 (63.3%) had moderate to severe disease on presentation and 11/30 (36.7%) had mild disease. These patients were referred to the psychiatry department of our institute for appropriate management of these sequelae. Possible risk factors such as sex, disease severity, hospitalization, ICU care, co-morbidities, etc. were correlated with these psychological sequelae and results have been tabulated in Tables 2, 4.

Persistent headache, prolonged ageusia, prolonged anosmia, and neuropathy were the neurological sequelae reported in 7/120 (5.8%), 6/120 (5%), 11/120 (9.2%), and 1/120 (0.8%) participants respectively. The correlation of neurological sequelae with possible risk factors like severity of disease, pre-existing co-morbidities like diabetes, smoking history, remdesivir use, etc. has been summarized in Tables 3, 4. Out of these 26 participants having neurological sequelae, 13 (50%) had mild disease, 4 (15.4%) had moderate disease and 9 (34.6%) had severe disease on presentation.

Eight out of 120 i.e., 6.6% of the study participants were diagnosed with new onset diabetes mellitus during the follow-up period based on their HbA1c levels (based on American diabetes association recommendations) with overall mean HbA1c rise from 5.9% on admission to 6.8% post six months of recovery. Out of eight who developed diabetes, six participants had received steroids during their acute Covid phase; none of the eight new onset diabetics suffered from a generalised anxiety disorder, a potential confounder.

Seven out of 120 (5.8%) of the participants developed mucormycosis during the follow-up period, and possible correlation was assessed with risk predictors like older age (d = 0.73), co-morbidities like diabetes (RR = 16.21; p <0.001), the severity of disease (relative risk {RR} = 7.84; p = 0.04), steroid use (RR = 23.93; p = 0.023) and obesity (d = 1.15). Risk association for all the systemic sequelae with continuous variables have been tabulated in Table 4.

## Discussion

A significant proportion of individuals who suffered from acute SARS-CoV-2 infection display a range of chronic symptoms that last for several months. Our study illustrated that not only patients having moderate to severe disease but even those with mild disease developed sequelae. Similar results have been observed in studies done in other parts of the world [8-10]. Zayet et al. who followed up on post-Covid cases of all severity classes found that up to 36% of their study participants had at least one persistent symptom after nine months post-recovery [10]. Since the onset of the pandemic, various terms have been used to describe this set of symptomatology, such as “post-acute sequelae of COVID-19” (PASC), “post-acute COVID-19 syndrome” (PACS), or “long-Covid” [11].

It has been found that patients with pre-existing co-morbidities, higher BMI, and older age are at a higher risk of developing severe disease [12]. In our observation, the severity of illness was a strong predictor of pulmonary (RR = 5.8846; p<0.001) or psychological (RR = 1.9112; p = 0.03) sequelae (Tables 1, 2). It implies that participants were about six times and two times more likely to develop pulmonary and psychiatric sequelae respectively. This can be attributed to the fact that patients who suffered from severe disease were prone to have residual lung parenchymal damage even after recovery, hence explaining the prolonged dyspnea and cough, as well as pulmonary fibrosis and recurrent pneumothorax. A study on post-Covid follow-up CT patterns showed that only 7% of cases had residual fibrotic abnormalities at the five-seven month mark which might depict a non-progressive self-resolving type of post-inflammatory fibrosis [13]. Patients who required positive pressure ventilation during Covid treatment (either invasive or non-invasive) were 19.8 times more likely to develop a pneumothorax later and 3.65 times more likely to develop pulmonary fibrosis (Refer to Table 1). It is difficult to ascertain whether the development of pulmonary fibrosis and pneumothorax is entirely due to inflammatory causes or at least partially due to barotrauma or volutrauma caused by positive pressure ventilation.

We however also observed that people with pre-existing co-morbidities like diabetes mellitus and hypertension were independently at a higher risk of developing pulmonary sequelae (RR = 1.7273; p = 0.019) regardless of the severity of the disease. Rather, 10/120 i.e., 8.3% of study participants having persistent post-covid respiratory symptoms had mild disease. Prolonged respiratory symptoms, especially dyspnea without significant hypoxia or structural lung damage may be explained as a psychosomatic symptom and these participants had a significant overlap with psychological sequelae, especially anxiety. Older age was also found to be significantly associated with the development of pulmonary and psychiatric sequelae like depression and PTSD.

Females were more predisposed to develop psychological sequelae as compared to males. The relative risk of the sequence in females compared to males was 1.9576, meaning that females were about two times as likely to develop these sequelae as compared to males. Observed relative risk was statistically significant (p=0.0248). This finding was similar to several other studies which indicate that female gender tends to be a consistent risk factor for psychiatric sequelae [14,15]. This can be attributed to the dimensional liability model of gender differences in mental disorder prevalence which shows that women have a higher mean level of internalizing (mood and anxiety) [16]. Also, the World Health Organization (WHO) reports overall female preponderance for anxiety/depression and gives an epidemiological basis for this finding [17]. Undernourished patients with lower BMI were also found to be at a higher risk to develop psychiatric sequelae like depression and GAD (Table 4). Risch et al. suggested that malnourishment has a strong association with psychiatric illnesses which may explain our observation [18]. Also, hospitalization for treating Covid had a significant association with psychological sequelae, with a RR of 6.009 (p = 0.001) showing that patients hospitalized for Covid treatment are six times more likely to develop psychiatric sequelae. The need for ICU care was especially associated with the diagnosis of post-traumatic stress disorder (PTSD) with RR = 86.7 and p = 0.001. Prolonged hospitalization and ICU experience take a tremendous toll on a patient's mental health. Witnessing deaths during hospitalization and experiencing that level of distress may contribute to developing psychological sequelae like dysthymia, depression, anxiety, and PTSD. Administration of medication like sedatives and paralytics, and experiencing mechanical ventilation can also have some impact on the psychological well-being of the patient. All these factors probably contributed to the psychological sequelae, but it is difficult to assess their role individually and hence have been clubbed together under “ICU stay” as a situational risk. Also, the financial burden of hospitalization has an additive impact on the patient's psychological well-being. The impact of such factors cannot be statistically measured with limited data size. Patients may even have a pre-existing undisclosed or undiagnosed psychiatric disorder which might be a confounding factor. These variables may have an indirect role in causing undue psychological distress to the patient and should be considered. Participants who received steroids for treatment of acute Covid during hospitalization were also found to be independently associated with the risk of developing psychological sequelae (RR = 1.82; p=0.04). Patients receiving corticosteroid treatment are known to develop psychiatric complications like psychosis and mood disorders [19]. Therefore, we may conclude that the use of steroids may have had an additive impact on participants who were hospitalized or were in ICU. However, the exact correlation between psychological sequelae and each possible risk factor individually could not be ascertained even in other studies with a larger sample size due to the overlap of multiple risk factors [14,15].

Study participants having a pre-existing diagnosis of bronchial asthma or allergic rhinitis were found to be at a higher risk of experiencing persistent anosmia (RR = 3.96) and ageusia (RR = 2.67). Chronically inflammed airway mucosal surface in these participants may attribute to this, but there is no such evidence reported in the literature. This association may require further observational studies and research.

A significant number of study participants (6.6%) were diagnosed with new onset diabetes mellitus during follow-up. Montefusco et al. illustrated that SARS-CoV-2 induces insulin resistance and abnormal beta cell function leading to aberrant glycemic control which persists in the post-acute phase of the illness [20]. Additionally, a momentary rise in HbA1c levels may be attributed to the administration of high-dose corticosteroids which may also disrupt glycemic control [21].

Mucormycosis occurred in 7/120 (5.8%) of our study participants and a significant association was found between risk predictors like diabetes (RR = 16.25), higher severity of Covid (RR = 7.84), older age (d = 0.73), obesity (d = 1.15) and corticosteroid use (RR = 23.93). Hyperglycemia has been found to be linked with the release of reactive oxygen species that causes tissue damage and thus increases the risk of mucormycosis development [22]. The use of steroids as a treatment for Covid also adversely impacts glycemic control and has also been shown to significantly increase the risk of mucormycosis in Covid [21]. Interestingly, all seven study participants who were diagnosed with mucormycosis on follow-up had received corticosteroids for more than two weeks during their treatment for Covid which highlights the strong association between liberal steroid use and the occurrence of opportunistic infections.

Out of 120 study participants, four patients (3.3%) died during the follow-up period. Three died a natural death, not directly related to Covid. The cause of death of one participant was not disclosed by the relatives. The post-Covid status in these individuals and underlying co-morbidities could have indirectly contributed to the mortality.

Strengths of our study include a stringent physical follow-up at well-defined intervals along with frequent telecommunications with the study participants and thorough evaluation for sequelae. Inclusion of all infected Covid cases irrespective of the case severity provided us with a fairly unbiased set of cohorts.

Limitations include self-reported details of symptomatology which are subjective and cannot be validated by medical/laboratory tests and a recall bias for symptoms reported from past memory. Also, as our study population mostly included middle-aged group and hence the findings cannot be extrapolated to older age groups or children.

## Conclusions

This study shows that the health effects of COVID-19 go well beyond a simple viral infection. The post-Covid after-effects encompass a wide range of symptomatology and may even lead to serious, even fatal complications. Risk factors associated with these sequelae can be patient-related (age, sex, co-morbidities, BMI) and treatment-related (hospitalization, ICU, steroid use). Detailed study of these risk factors would help clinicians to monitor for specific sequelae in future cases. A post-Covid follow-up is warranted in all patients, especially those with these risk factors, for appropriate rehabilitation.
